# Gastric metastasis from high-grade soft tissue sarcoma: a rare occurrence with literature review

**DOI:** 10.1093/jscr/rjaf192

**Published:** 2025-04-04

**Authors:** Blagica Krsteska, Tamara Angelovska, Panche Zdravkovski, Xhem Adem, Teodora Todorova, Milan Samardziski, Darko Dzambaz, Slavica Kostadinova-Kunovska

**Affiliations:** Faculty of Medicine, Institute of Pathology, University Ss. Cyril and Methodius in Skopje, 50 Divizija No.6, Skopje 1000, Republic of North Macedonia; Faculty of Medicine, Institute of Pathology, University Ss. Cyril and Methodius in Skopje, 50 Divizija No.6, Skopje 1000, Republic of North Macedonia; Faculty of Medicine, Institute of Pathology, University Ss. Cyril and Methodius in Skopje, 50 Divizija No.6, Skopje 1000, Republic of North Macedonia; University Clinic of Gastroenterohepatology, Mother Teresa No.17, Skopje 1000, Republic of North Macedonia; University Clinic for Orthopedic Surgery, Mother Teresa No.17, Skopje 1000, Republic of North Macedonia; University Clinic for Orthopedic Surgery, Mother Teresa No.17, Skopje 1000, Republic of North Macedonia; University Clinic for Digestive Surgery, Mother Teresa No.17, Skopje 1000, Republic of North Macedonia; Faculty of Medicine, Institute of Pathology, University Ss. Cyril and Methodius in Skopje, 50 Divizija No.6, Skopje 1000, Republic of North Macedonia

**Keywords:** gastric metastasis, sarcoma, pleomorphic sarcoma, melena, anemia

## Abstract

Metastasis of high-grade soft tissue sarcoma to the stomach is an extremely rare occurrence. While sarcomas can spread to distant organs, they most commonly metastasize to the lungs, liver, and bones. We report a unique case of gastric metastasis from a high-grade soft tissue sarcoma, occurring 4 years after the initial diagnosis of fibrosarcoma in the right femoral region. The patient did not undergo adjuvant chemotherapy and developed a large soft tissue metastasis in the left gluteal region 2 years later. After 4 years, he presented with a second soft tissue metastasis and suspected metastatic lung nodules. Symptoms of melena and severe anemia prompted a gastroscopic examination, which revealed gastric metastasis from a high-grade soft tissue sarcoma. Due to severe anemia, a palliative gastric resection was performed. A review of the literature indicates that metastatic leiomyosarcomas are the most frequently reported sarcoma subtype metastasizing to the stomach.

## Introduction

High-grade soft tissue sarcoma metastasizing to the stomach is exceedingly rare. Sarcomas do metastasize to distant organs, but the most common sites of spread include the lungs, liver, and bones. Metastatic involvement of the stomach is very uncommon, and only a few cases have been reported in the literature [1–9]. This rarity could be related to the peculiar vascular and lymphatic features of the gastric mucosa, which may not be propitious for the implantation of tumor cells. When gastric metastases do occur, they are most often incidentally found during imaging or endoscopic studies, since they might be asymptomatic or mimic primary gastric malignancies. Because it is a rare condition, clinicians may not consider gastric metastasis in patients with soft tissue sarcoma, which delays diagnosis and treatment. This case report and literature review try to increase awareness of this infrequent metastatic pattern. To our knowledge, this is the first case of high-grade soft tissue sarcoma gastric metastasis in our country and the second published in the literature.

## Case report

**Figure 1:**
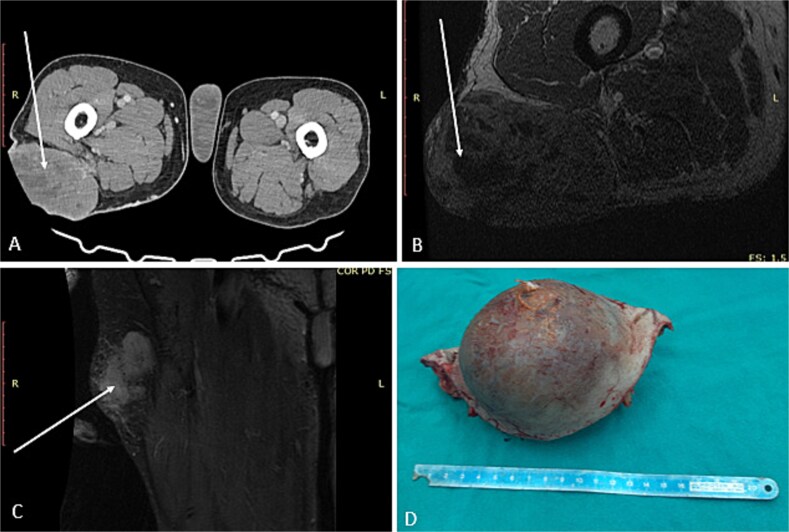
First surgical treatment. (A) CT, (B and C) MRI, (D) surgical specimen.

А 68-year-old male presented at the University Clinic for Orthopedic Surgery with a soft tissue mass at the right femoral region. After detailed clinical and radiological investigations, the tumor board decided that a wide resection was most beneficial (Fig. 1A–C). Grossly the tumor measured 10 × 8.6 × 7.5 cm, white to tan colored tumor tissue with a soft consistency and large areas of necrosis and hemorrhage, surrounded with a pseudocapsule. The tumor was covered with intact and elevated skin (Fig. 1D). Microscopy revealed a highly cellular tumor composed of elongated, spindle cells in a “herringbone” arrangement with collagen deposition between cells. Large areas of hemorrhage and necrosis were confirmed. The tumor cells were negative for S-100, desmin, and smooth muscle actin (SMA), while positive for vimentin. A diagnosis of fibrosarcoma was made, and according to UICC, 8th edition the disease was in the IIIB stage. After the surgical treatment, the patient refused the course of adjuvant chemotherapy, and 2 years later the patient presented again with a large soft tissue mass in the left gluteal region confirmed as metastasis.

Computer tomography (CT) of the thorax, abdomen, and pelvis revealed infiltrative lesions in the lungs highly suspected for secondary deposits. The patient refused adjuvant chemotherapy for the second time.

**Figure 2:**
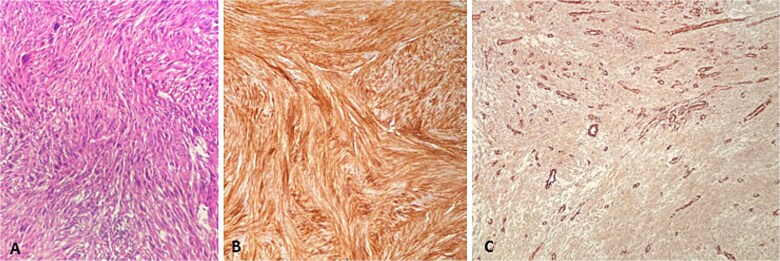
Second metastasis from high grade soft tissue sarcoma. (A) HE ×200, (B) CD10 ×200, (C) SMA ×100.

**Figure 3:**
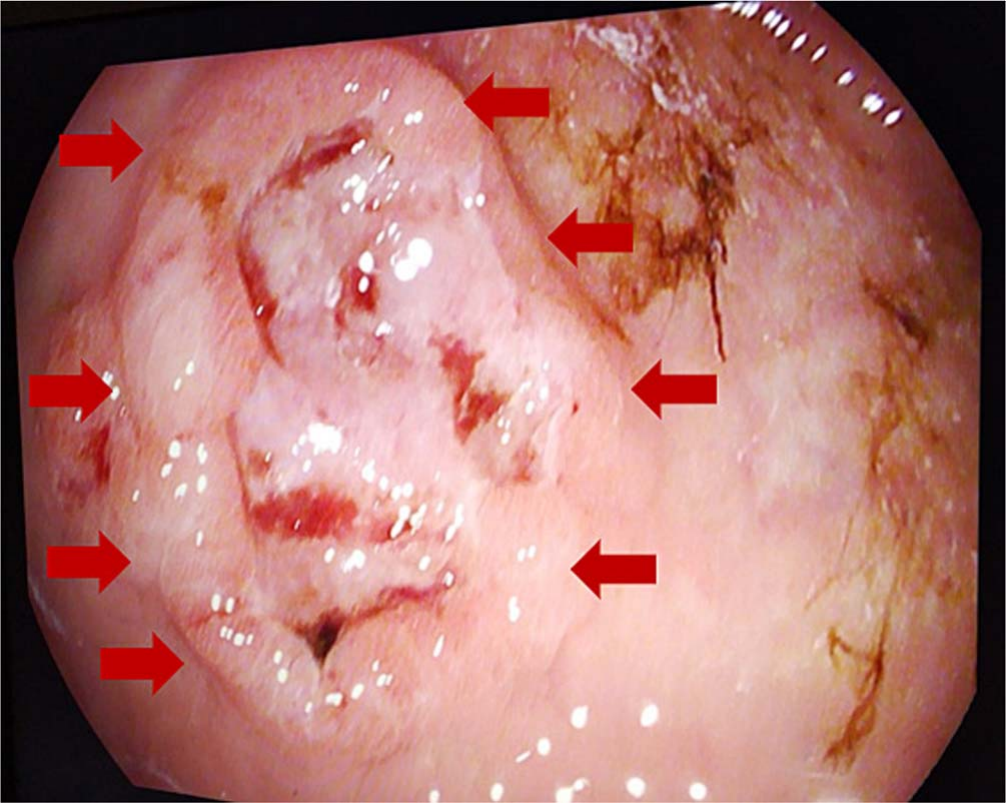
Endoscopic gastric ulcerative lesion.

The patient presented for the third time, after 4 years of initial diagnosis, with a soft tissue mass in the right gluteal and posterior femoral region. Wide resection was performed revealing two tumor masses. The tumor of the right gluteal region weighted 21 g and measured 4 × 3.5 × 2.5 cm with surrounding fat tissue. On cross-section, the tumor measured 2.5 cm. Microscopically, it was composed predominantly of spindled cells in a herringbone pattern, with areas of necrosis and hemorrhage, as well as larger, more pleomorphic cells with hyperchromatic nuclei. The resection from the posterior femoral region weighed 235 g, measuring 12.5 × 6.5 × 5.5 cm, containing a 9.5 × 4.5 × 5 cm tumor surrounded by skeletal muscle. The tumor was with the described morphology and presence of a high grade pleomorphic component. Immunohistochemistry showed a diffuse CD10 positivity. The tumor cells were negative for Desmin, SMA, Caldesmon, and CD56 (Fig. 2) During the postoperative period, he was continuously anemic Er 2.6 × 10^12^/L; hemoglobin 81 g/L; hematocrit 0.23|L/L, which indicated further investigations. Gastroscopy was performed and an ulcerative lesion was measuring 2 cm located on the front gastric wall near the great curvature (Fig. 3).

**Figure 4:**
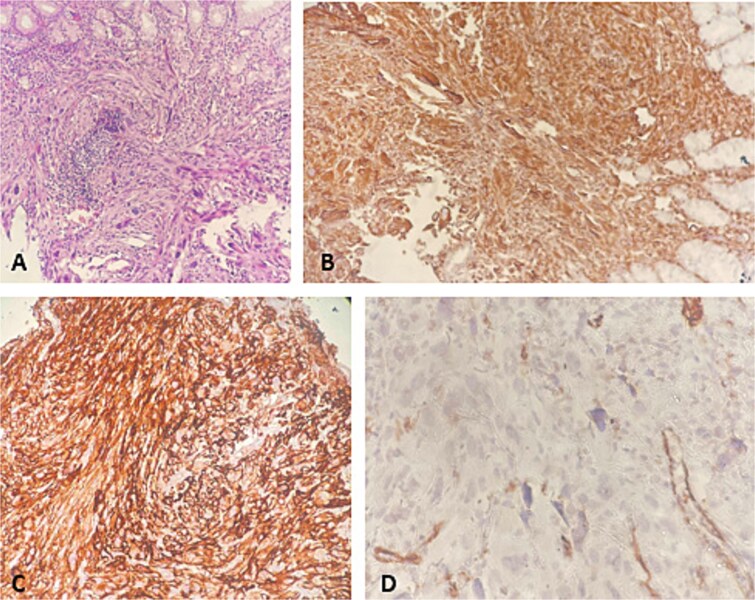
Gastric biopsy. (A) HE ×200; (B) vimentin ×200; (C) CD10 ×200; (D) SMA ×200.

**Figure 5:**
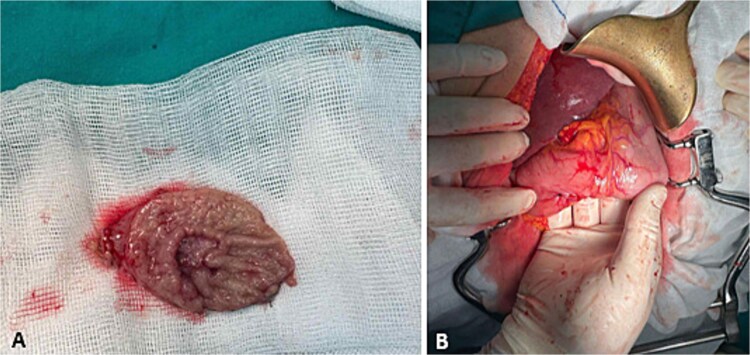
Gastric surgery. (A) Wedge gastric resection; (B) suture after gastric resection.

**Figure 6:**
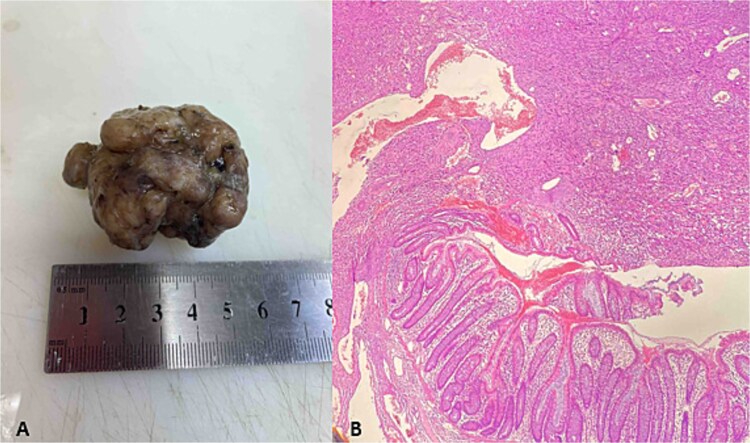
Polypoid metastasis in right colon. (A) Gross specimen; (B) HE×100.

Gastric biopsies revealed tumor infiltration of pleomorphic tumor cells with bizarre nuclei and mitotic figures that showed positivity only for Vimentin and CD10, and a diagnosis of metastatic high grade sarcoma was made (Fig. 4). Due to blood loss and severe anemia the patient was admitted to University Clinic for abdominal surgery. Bimanual palpation determined the lesion to be localized to the grater curvature of the stomach at the transition from the corpus to the antrum. A wedge resection was made of the segment that contained the lesion with a GIA stapler without compromising the permeability of the stomach. The post-operative course was settled and quickly established per os feeding (Fig. 5). One week after the resection, due to symptoms of melena, the patient was investigated by colonoscopy where a polypoid lesion measuring 4.5 cm was found in the right colon (coecum) (Fig. 6). It was surgically removed by a short right colectomy, and histology confirmed as high grade sarcoma metastasis. To the best of our knowledge, this is the first case of gastric and colon metastasis from soft tissue high-grade sarcoma.

### Discussion

Understanding the patterns of sarcoma metastatic sites is crucial for accurate staging, prognosis, and treatment planning. Gastric metastasis is rare, but it happens when a tumor in one part of the body spreads and deposits cancer cells into the stomach. The stomach is one of the more frequent sites of metastasis for melanoma, breast tumors, and lung tumors [10, 11]. Sarcomas typically metastasize to the lungs, liver, and bones; however, gastric metastasis is exceedingly rare. The patterns of sarcoma spread to the stomach are not well-defined due to the limited number of reported cases [12, 13]. Gastric metastases are typically identified during evaluations for gastrointestinal symptoms. Endoscopic procedures with biopsy are essential for accurate diagnosis. Treatment options are primarily palliative, focusing on symptom relief. Surgical interventions, such as metastasectomy, may be considered to address complications like bleeding or obstruction, aiming to improve the patient's quality of life.

Palliative chemotherapy, targeted therapy, or immunotherapy may be indicated depending on what type of the primary cancer is present and the level of metastasis. The prognosis is mostly unsatisfactory due to the late stage of disease when it is diagnosed. Sarcoma metastasis in the stomach is an extremely rare occurrence, briefly presented in Table 1 as published in the literature.

**Table 1:** Published cases of sarcoma gastric metastasis.

**Year**	**Author**	**Gender, age (years)**	**Primary localization**	**Initial diagnosis**	**Time period of gastric metastasis**	**Type of gastric resection/treatment**	**Other metastases**
2006	Doddis *et al.* [1]	Female, 72	Left femur	Ewing sarcoma	6 years	3 hemostatic clips, epinephrine solution	/
2006	Akatsu *et al.* [2]	Male, 75	Left side on the back	Malignant fibrous hystiocytoma	2 years	Partial gastric resection	/
2010	Dent *et al.* [3]	Male,60	Left posterior shoulder	Soft tissue sarcoma	2 weeks	Snare and cautery	Lung
2012	Yodonawa *et al.* [4]	Male, 73	Kidney	Leiomyosarcoma	2 years	Distal gastrectomy	Lung, liver, bone
2013	Urakawa *et al.* [5]	Male, 73	Sternum	Osteosarcoma	11 months	Partial gastrectomy	Lung
2016	Samuel *et al.* [6]	Male, 56	Left thigh	Synovial sarcoma	1 year	Chemotherapy	Lung, liver
2019	Koti *et al.* [7]	Female, 14	Right femur	Ewing sarcoma	/	Chemotherapy, total gastrectomy	/
2021	Eiswerth *et al.* [8]	Male, 61	Lung	Leiomyosarcoma	At the time of diagnosis	Declined chemotherapy and radiation	Duodenum
2023	Uchiyama *et al.* [9]	Female, 59 Male, 4	Right thigh Left leg	Leiomyosarcoma	9 years 2 years	Partial gastrectomy, distal pancreatectomy, Combined gastric resection	Lung, bone, pancreas, Lung, bone
2025	Current case	Male, 68	Right femoral region	Soft tissue sarcoma	4 years	Wedge gastric resection	Lung, colon (coecum)

Gastric metastasis from high-grade soft tissue sarcomas is very rare. Soft tissue sarcomas (STS) are ˂1% of all cancers, with leiomyosarcoma (LMS) making up 10%–20% of these. The most common places for STS to spread are the lungs, liver, and bones, while stomach involvement is uncommon. A recent study reported two LMS cases with gastric metastasis [9]. In both cases, the metastases were found as single lesions in the upper anterior wall and the body-greater curvature of the stomach. The patients had surgery to remove the metastases due to problems like stomach bleeding, abdominal pain, and trouble swallowing, with the aim of improving their quality of life.

Another case involved a patient with a high-grade pleomorphic sarcoma that started as a mass in the left shoulder area. After surgery, the patient showed gastrointestinal issues, and an endoscopy later found a metastatic lesion in the stomach [3]. Despite treatment, the patient's health worsened because of widespread metastasis.

There is also a case of Ewing's sarcoma in the right proximal femur that spread to the stomach in a young woman [7]. This highlights the unpredictable nature of how sarcomas spread. Because cases like these are so rare, there is not much agreement on standard treatments for gastric metastasis from high-grade soft tissue sarcomas. Management is often tailored to the individual, focusing on relieving symptoms and improving quality of life. Surgical options may be considered in some cases, especially when addressing issues like bleeding or blockage.

## Conclusion

Although rare, gastric metastasis should be considered when gastrointestinal symptoms or anemia occur in patients with diagnosed sarcomas.

## References

[1] Dodis LB, Bennett MW. Ewing’s sarcoma metastasis to the gastric wall in a 72-year-old patient. MedGenMed 2006;8:6. PMC1781288 17406148

[2] Akatsu Y, Saikawa Y, Kubota T, et al. Metastatic gastric cancer from malignant fibrous histiocytoma: report of a case. Surg Today 2006;36:385–9. 10.1007/s00595-005-3163-8. 16554998

[3] Dent LL, Cardona CY, Buchholz MC, et al. Soft tissue sarcoma with metastasis to the stomach: a case report. World J Gastroenterol 2010;16:5130–4. 10.3748/wjg.v16.i40.5130. 20976852 PMC2965292

[4] Yodonawa S, Ogawa I, Yoshida S, et al. Gastric metastasis from a primary renal leiomyosarcoma. Case Rep Gastroenterol 2012;6:314–8. 10.1159/000338837. 22754492 PMC3376334

[5] Urakawa H, Tsukushi S, Tsurudome I, et al. Metastasis of osteosarcoma to stomach made clinically evident by hematemesis: a case report. World J Surg Oncol 2013;11:48. 10.1186/1477-7819-11-48. 23442337 PMC3599039

[6] Samuel T, Norly S, Ros’aini P. Gastric ulcer that turned out to be metastasis of a synovial sarcoma: a case report and literature review. Med J Malaysia 2016;71:363–5. 28087966

[7] Koti KA, Backianathan S, Sebastian P, et al. A rare case of gastric metastasis in Ewing’s sarcoma of the femur. Case Rep Oncol Med 2019;2019:2870302. 10.1155/2019/2870302. 31218087 PMC6537017

[8] Eiswerth MJ, Pinter A, Reynolds SB, et al. Primary lung sarcoma with gastric metastasis and morphological divergence presenting as melena. BMJ Case Reports CP 2021;14:e242364. 10.1136/bcr-2021-242364. PMC8362720 34385220

[9] Uchiyama T, Nakamura T, Nakata K, et al. Gastric metastasis in patients with leiomyosarcoma: a case report. Biomed Rep 2023;19:1–6. 10.3892/br.2023.1657 PMC10511945 37746592

[10] Altay AY, Buyuk M, Ozgur I, et al. Metastases to the stomach: clinicopathologic features of metastases mimicking gastric primaries. *Turkish Journal of Pathology* 2021:37;203–11. 10.4183/aeb.2021.521 PMC10510621 34514560

[11] Ibrahimli A, Aliyev A, Majidli A, et al. Metastasis to the stomach: a systematic review. F1000Res 2023;12:1374. 10.12688/f1000research.140758.1. 38706640 PMC11066534

[12] WHO Classification of Tumours . Digestive System Tumours (5ed.). Lyon: IARC, 2019.

[13] WHO Classification of Tumours . Soft tissue and bone tumours. Lyon (France): International Agency for Research on Cancer; 5ed. Vol. 3;2020. 10.1001/jamanetworkopen.2020.26312

